# REDD1 Is Involved in Amyloid β-Induced Synaptic Dysfunction and Memory Impairment

**DOI:** 10.3390/ijms21249482

**Published:** 2020-12-13

**Authors:** Jee Hyun Yi, Huiyoung Kwon, Eunbi Cho, Jieun Jeon, Jeongwon Lee, Young Choon Lee, Jong Hyun Cho, Mira Jun, Minho Moon, Jong Hoon Ryu, Ji-Su Kim, Ji Woong Choi, Se Jin Park, Seungheon Lee, Dong Hyun Kim

**Affiliations:** 1 Center for Synaptic Brain Dysfunctions, Institute for Basic Science, Daejeon 34141, Korea; jeehyunyi@kaist.ac.kr; 2 Department of Health Sciences, The Graduate School of Dong-A University, Dong-A University, Busan 49315, Korea; kwonhuiyoung@naver.com (H.K.); bee2634@naver.com (E.C.); ji6785@naver.com (J.J.); yclee@dau.ac.kr (Y.C.L.); jhcho1@dau.ac.kr (J.H.C.); mjun@dau.ac.kr (M.J.); 3 Department of Marine Life Science, Jeju National University, Jeju 63241, Korea; liar0510@naver.com; 4 Department of Biochemistry, College of Medicine, Konyang University, Daejeon 35365, Korea; hominmoon@konyang.ac.kr; 5 Department of Oriental Pharmaceutical Science, College of Pharmacy, Kyung Hee University, Seoul 02447, Korea; jhryu63@khu.ac.kr; 6 Primate Resources Center (PRC), Korea Research Institute of Bioscience and Biotechnology (KRIBB), Jeongup-si, Jeollabuk-do 56216, Korea; kimjs@kribb.re.kr; 7 College of Pharmacy and Gachon Institute of Pharmaceutical Sciences, Gachon University, Incheon 21936, Korea; pharmchoi@gachon.ac.kr; 8 School of Natural Resources and Environmental Sciences, Kangwon National University, Chuncheon 24341, Korea; sejinpark@kangwon.ac.kr; 9 Institute of Convergence Bio-Health, Department of Health Sciences, The Graduate School of Dong-A University, Busan 49315, Korea

**Keywords:** Alzheimer’s disease, REDD1, Aβ, hippocampal long-term potentiation, learning and memory

## Abstract

Alzheimer’s disease (AD) is a neurodegenerative disease characterized by neurological dysfunction, including memory impairment, attributed to the accumulation of amyloid β (Aβ) in the brain. Although several studies reported possible mechanisms involved in Aβ pathology, much remains unknown. Previous findings suggested that a protein regulated in development and DNA damage response 1 (REDD1), a stress-coping regulator, is an Aβ-responsive gene involved in Aβ cytotoxicity. However, we still do not know how Aβ increases the level of REDD1 and whether REDD1 mediates Aβ-induced synaptic dysfunction. To elucidate this, we examined the effect of Aβ on REDD1-expression using acute hippocampal slices from mice, and the effect of REDD1 short hairpin RNA (shRNA) on Aβ-induced synaptic dysfunction. Lastly, we observed the effect of REDD1 shRNA on memory deficit in an AD-like mouse model. Through the experiments, we found that Aβ-incubated acute hippocampal slices showed increased REDD1 levels. Moreover, Aβ injection into the lateral ventricle increased REDD1 levels in the hippocampus. Anisomycin, but not actinomycin D, blocked Aβ-induced increase in REDD1 levels in the acute hippocampal slices, suggesting that Aβ may increase REDD1 translation rather than transcription. Aβ activated Fyn/ERK/S6 cascade, and inhibitors for Fyn/ERK/S6 or mGluR5 blocked Aβ-induced REDD1 upregulation. REDD1 inducer, a transcriptional activator, and Aβ blocked synaptic plasticity in the acute hippocampal slices. REDD1 inducer inhibited mTOR/Akt signaling. REDD1 shRNA blocked Aβ-induced synaptic deficits. REDD1 shRNA also blocked Aβ-induced memory deficits in passive-avoidance and object-recognition tests. Collectively, these results demonstrate that REDD1 participates in Aβ pathology and could be a target for AD therapy.

## 1. Introduction

Alzheimer’s disease (AD) is the most common neurodegenerative disease associated with memory and cognitive impairment [1,2]. Although AD is recognized as a global health problem, and various pathological mechanisms have been revealed, appropriate medicine is yet to be developed due to the complex pathogenesis [3,4,5]. Therefore, new targets for preventing AD are urgently needed.

A stress-inducible protein is “regulated in development and DNA damage response 1” (REDD1), also known as RTP801 or Dig2, which is upregulated in response to a variety of cellular stresses such as nutrient and energy deprivation [6,7], hypoxia [8], DNA damage [9], and stress hormone glucocorticoids [10,11]. REDD1, a negative regulator of the mammalian target of rapamycin (mTOR), is involved in transcription and modulates Akt activity by suppressing mTOR via tuberous sclerosis complex 1 (TSC1)/tuberous sclerosis complex 2 (TSC2), and inactivation of Ra homolog enriched in brain (Rheb) [8]. Because mTOR is involved in diverse phenomena, such as autophagy [10], cell proliferation [6], and cell motility [12], its regulation by REDD1 has the potential to be a pharmacological target for various neurological diseases. Metformin, a widely prescribed Type 2 diabetes drug, was found to induce mTOR inhibition and cell-cycle arrest through REDD1 [13].

REDD1 is closely associated with neurological diseases because mTOR is a crucial protein that regulates synapse formation and plasticity [14,15]. Hence, an increase in REDD1 expression was observed in patients with Parkinson’s disease [16], and dopaminergic neurotoxin 6-OHDA upregulated REDD1 in vitro [17] and in vivo [18]. Moreover, *DDIT4*, a REDD1 gene, is a gene responding to amyloid β (Aβ), a pathological hallmark of Alzheimer’s disease [19]. Additionally, it acts as a critical mediator of stress-induced synaptic loss and depressive behavior [20]. Previous findings indicated that REDD1 is upregulated by Aβ, and the antisense of *DDIT4* inhibits Aβ cytotoxicity [19]. However, we still do not know how Aβ increases REDD1 levels and how REDD1 is involved in Aβ toxicity. To elucidate this, we examined the mechanism of Aβ-induced REDD1 upregulation and the role of REDD1 in Aβ-induced synaptic deficits using acute hippocampal slices from mice. Lastly, we examined the effect of REDD1 knockdown in memory deficits in AD-like mice models.

## 2. Results

### 2.1. Aβ Increased REDD1 Levels in the Hippocampus

**Figure 1:**
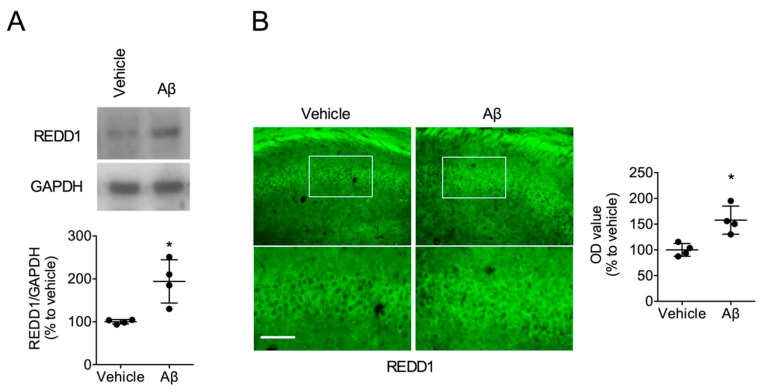
Amyloid β (Aβ) increased regulated in development and DNA damage response 1 (REDD1) protein levels in the hippocampus. (**A**) Aβ-induced REDD1 upregulation. Hippocampal slices were incubated with Aβ for 4 h. (**B**) Aβ-induced REDD1 upregulation in the hippocampus. Aβ was injected into the fissure layer of the hippocampal CA1 region. Bar = 50 μm. Data represented as mean ± SD with raw data. * *p* < 0.05 vs. vehicle-treated group.

To examine whether Aβ regulates REDD1 expression, we tested the REDD1 levels in Aβ-treated hippocampal slices from mice. The hippocampal slices, treated with Aβ (1 μM) for 4 h, showed significantly increased REDD1 levels (t_6_ = 7.802, *p* < 0.05, n = 4/group; Figure 1A). Moreover, intracerebroventricular injection of Aβ (10 μM, 3 μL) into the hippocampal CA1 region increased REDD1 levels 24 h postinjection (t_6_ = 3.871, *p* < 0.05, n = 4/group; Figure 1B). These results suggest that Aβ may upregulate REDD1.

### 2.2. Fyn/ERK/S6 Signaling Is Involved in Aβ-Induced REDD1 Translation

**Figure 2:**
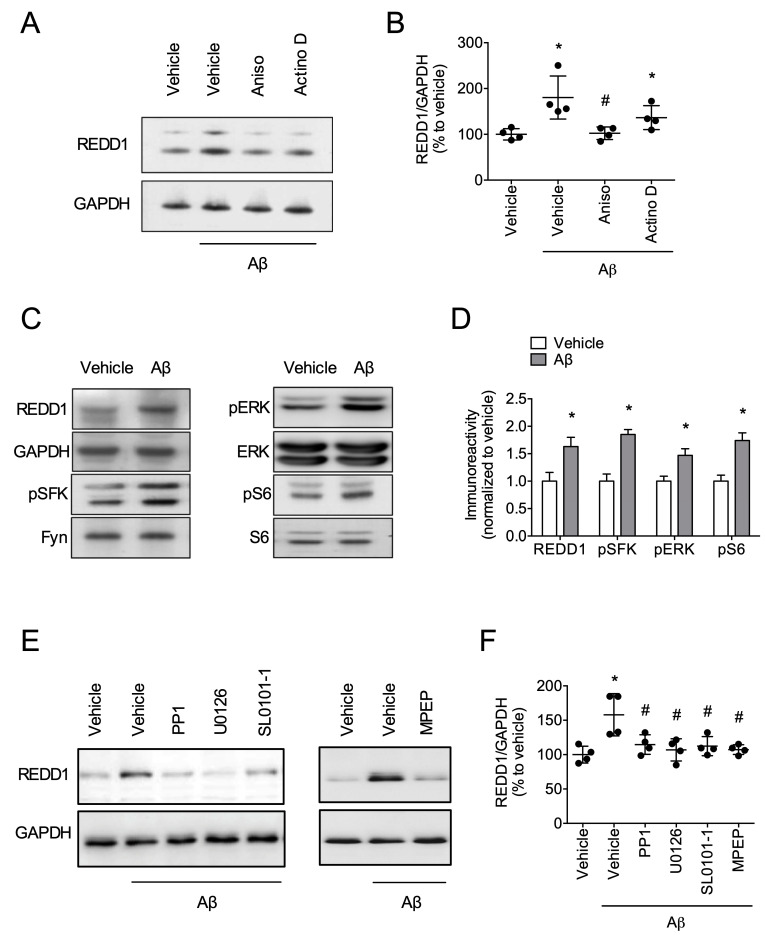
Fyn/ERK/S6 signaling is involved in Aβ-induced REDD1 translation. (**A**,**B**) Aβ increased REDD1 with translational modulation. Hippocampal slices were incubated with Aβ for 4 h with or without anisomycin (40 μM) or actinomycin D (50 μM). (**C**,**D**) Aβ activated Fyn/ERK/S6 signaling. Hippocampal slices were incubated with Aβ for 4 h. (**E**,**F**) Fyn/ERK/S6 signaling is required for Aβ-increased REDD1. Hippocampal slices were incubated with Aβ for 4 h with or without PP1 (10 μM), U0126 (50 μM), SL0101-1 (50 μM), or MPEP (10 μM). Data represented as mean ± SD with raw data. * *p* < 0.05 vs. vehicle-treated group. # *p* < 0.05 vs. Aβ-treated group.

To test whether Aβ affects REDD1 transcription or translation, we tested anisomycin and actinomycin D in the acute hippocampal slices. In this experiment, anisomycin, but not actinomycin D, blocked Aβ-induced upregulation of REDD1 (Figure 2A,B), suggesting that Aβ regulated the translation of REDD1. Previous studies suggested that Aβ affected Fyn/ERK/S6 signaling, which is involved in protein translation [21]. Therefore, we tested whether this signaling was also involved in Aβ-induced REDD1 overexpression in the hippocampal tissue. Aβ (1 μM for 4 h) significantly increased Fyn/ERK/S6 signaling in the hippocampus (Figure 2C,D). Next, to test whether this signaling is required for Aβ-induced upregulation of REDD1, we tested inhibitors of these molecules in the hippocampal tissue. PP1, a Fyn inhibitor; U0126, an ERK inhibitor; SL0101-1, a S6 inhibitor; and MPEP, an mGluR5 inhibitor blocked Aβ-induced upregulation of REDD1 in the hippocampal tissue (Figure 2E,F). These results suggest that Fyn/ERK/S6 signaling is involved in Aβ-induced upregulation of REDD1.

### 2.3. REDD1 Is Required for Aβ-Induced Synaptic Dysfunction

**Figure 3:**
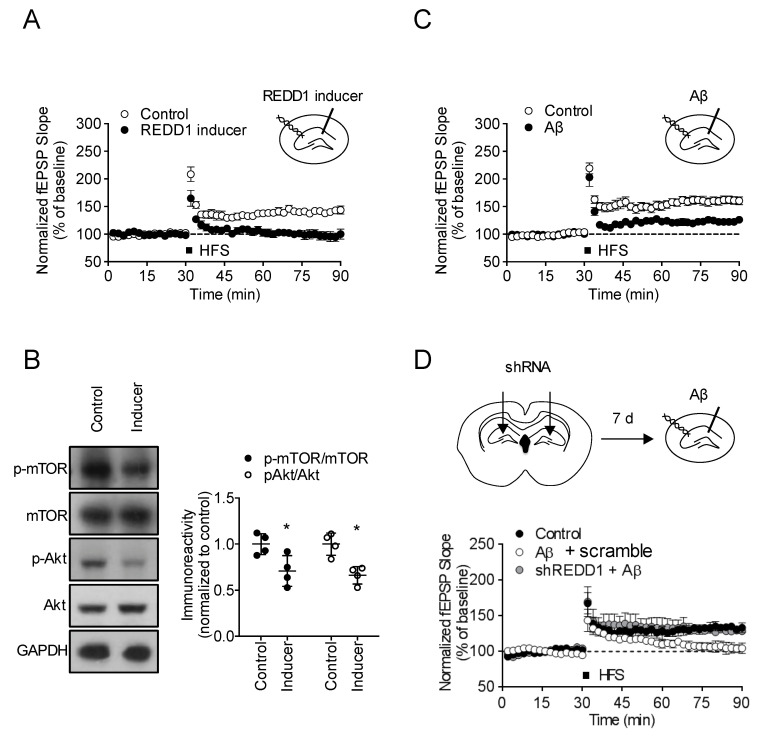
REDD1 is required for Aβ-induced synaptic dysfunction. (**A**) REDD1 inducer suppressed hippocampal long-term potentiation (LTP). Hippocampal slices incubated with REDD1 inducer (50 μM) for 4 h. Data represented as mean ± SD. (**B**) REDD1 inducer suppressed mTOR signaling in the hippocampus. Data represented as mean ± SD with raw data. (**C**) Aβ suppressed hippocampal LTP. Hippocampal slices incubated with Aβ (1 μM) for 4 h. Data represented as mean ± SD. (**D**) REDD1 knockdown blocked Aβ-induced LTP impairment. REDD1 shRNA (m) lentiviral particle or scramble lentiviral particle was bilaterally injected into hippocampal fissure layer. Hippocampal slices prepared and incubated with Aβ for 4 h, 7 d after lentiviral injection. Data represented as mean ± SD.

To determine if REDD1 is a mediator of Aβ-induced synaptic dysfunction, we tested the effects of a REDD1 inducer, a transcriptional inducer [22,23], and REDD1 shRNA in synaptic plasticity. REDD1 inducer (50 μM for 4 h) blocked high-frequency stimulation (HFS)-induced long-term potentiation (LTP) induction (*t*_8_ = 3.737, *p* < 0.05, n = 5/group; Figure 3A). Moreover, REDD1 inducer decreased mTOR/Akt signaling in the hippocampal slices (Figure 3B). These results suggest that REDD1 activation negatively regulates synaptic plasticity and inhibits mTOR/Akt signaling.

Next, to test whether REDD1 is required for Aβ-induced synaptic dysfunction, we tested the effect of REDD1 shRNA on Aβ-induced synaptic dysfunction. Aβ (1 μM for 4 h), which was aggregated for 24 h, blocked hippocampal LTP, which is induced by HFS (*t*_8_ = 3.901, *p* < 0.05, *n* = 5/group; Figure 3C). Seven days after the injections of REDD1 shRNA, hippocampal slices from mice were prepared for electrophysiology (Figure 3D). Aβ treatment significantly suppressed LTP levels in the scramble-treated hippocampal slices. In the short hairpin REDD1 (shREDD1)-injected hippocampal slices, Aβ failed to suppress LTP levels, suggesting that REDD1 is required for Aβ-induced synaptic deficit (*F*_2,12_ = 7.709, *p* < 0.05, *n* = 5/group; Figure 3D).

### 2.4. REDD1 Knockdown Rescued Aβ-Induced Memory Impairments

**Figure 4:**
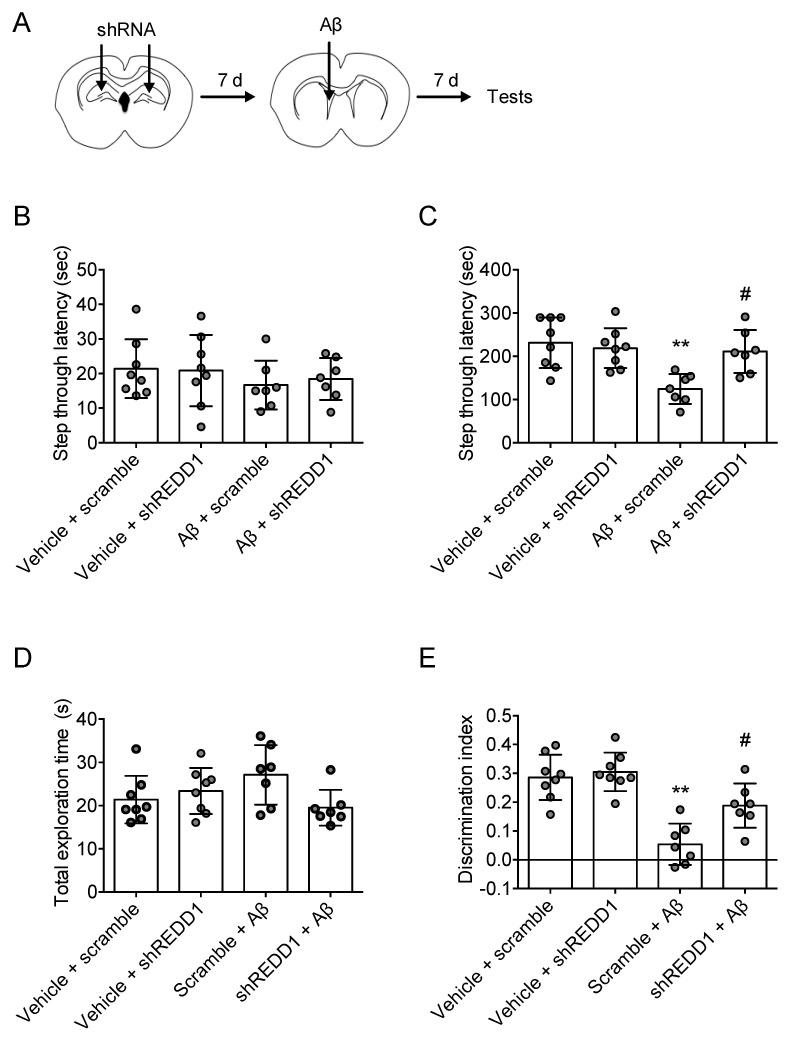
REDD1 reduction rescued Aβ-induced memory impairment. (**A**) REDD1 shRNA (m) lentiviral particle or control lentiviral particle was bilaterally injected into hippocampal fissure layer. Aβ was injected into the lateral ventricle 7 d later than the shRNA injection was. (**B**,**C**) REDD1 knockdown blocked Aβ-induced passive-avoidance memory deficit. Data represented as mean ± SD. ** *p* < 0.01 vs. sham group; # *p* < 0.05 vs. scramble + Aβ group. (**D**,**E**) REDD1 knockdown blocked Aβ-induced object-recognition memory deficit. Data represented as mean ± SD. # *p* < 0.05. ** *p* < 0.01.

To determine whether REDD1 is involved in Aβ-induced memory impairments, we used an intracerebroventricular injection of the Aβ model [24]. Aβ was injected into the lateral ventricle 7 days after shREDD1 injection. Behavioral tests were conducted 7 days after Aβ injection (Figure 4A). In the passive-avoidance test, shREDD1 or Aβ injection did not affect step-through latency in the acquisition trial (F_2,19_ = 0.7345, *p* > 0.05, *n* = 7–8/group; Figure 4B). In the test trial of the passive-avoidance test, Aβ reduced step-through latency in scramble-injected mice, but not in shREDD1-injected mice (F_2,19_ = 7.141, *p* < 0.05, *n* = 7–8/group; Figure 4C). The object-recognition test revealed no significant difference in total exploration time (F_2,19_ = 0.794, *p* > 0.05, n = 7–8/group, Figure 4D). Discrimination index showed that Aβ impaired recognition memory, and shREDD1 blocked this impairment (*F*_3,26_ = 17.95, *p* < 0.05, *n* = 7–8/group; Figure 4E). These results suggest that REDD1 knockdown abolished Aβ-induced memory loss.

## 3. Discussion

In the present study, REDD1, an mTORC1 repressor, was found to be upregulated by Aβ, which requires Fyn/ERK/S6 cascade. REDD1 is required for Aβ-induced synaptic deficit and memory loss. REDD1 blocked memory loss in an AD-like mouse model, suggesting that REDD1 could be a potential pharmacological target for memory loss in AD patients.

REDD1 is an upstream repressor of mTORC1 signaling and is upregulated in response to various stressors [25,26,27]. REDD1 expression is induced by protein expression as part of the endoplasmic reticulum stress response, including activating transcription factor 4 (ATF4) [28]. Upregulation of REDD1 was found in an AD brain [29,30]. However, the mechanism of upregulation of REDD1 and its role in AD are yet to be elucidated. Aβ increased the mRNA levels of REDD1, and the antisense REDD1 gene blocked Aβ cytotoxicity [19]. In the present study, we found that REDD1 is required for Aβ synaptotoxicity and AD-like memory impairment.

REDD1 is also upregulated in other brain diseases, including major depressive disorder [20,27]. REDD1 is required for stress-induced synaptic loss and depressive behavior. This process requires mTOR suppression-induced repression of translation of synaptic proteins, which results in basal synaptic deficit. In the present study, REDD1 activator suppressed hippocampal LTP in the Shaffer collateral pathway, suggesting that REDD1 suppresses either basal synaptic functions or synaptic plasticity. REDD1 suppresses mTORC1 [31,32]. Various synaptic stimulations, including glutamate and neurotrophins, activate mTORC1, thereby stimulating protein translation-induced changes in the synapse [33,34,35]. mTORC1 induces translocation of the AMPA receptor to the synaptic region via the S6K1 pathway [36,37]. PERK, mTORC1, and eEF2 interact during LTP induction [38]. These studies demonstrated that REDD1 upregulation could induce synaptic dysfunction through mTORC1 suppression, and this may be a mechanism of synaptic deficit in various stressful conditions of the brain, including AD. In the present study, we found that Aβ upregulated REDD1. Suppression of REDD1 expression with shRNA blocked Aβ-induced synaptic plasticity impairment, suggesting that REDD1 is a mediator of Aβ synaptotoxicity.

Controversial data were obtained regarding the role of mTOR in AD. In AD patients, mTORC1 was upregulated in the brain [39]. In Tg2576 mice, mTOR knockdown reduced amyloid deposits and ameliorated memory impairment [40]. Rapamycin, an mTOR inhibitor, decreased amyloid deposits and tau tangles, and reduced cognitive deficits in 3xTg and PDAPP mice [41,42]. However, several studies reported the downregulation of mTOR signaling in the Tg2576 model [43,44]. This could be due to differences in different mTOR complexes, including mTOR complex 1 (mTORC1) and complex 2 (mTORC2). The mTORC1 complex plays a critical role in synaptic plasticity [45,46,47]. However, the precise role of mTORC2 is yet to be elucidated. Several studies revealed that mTORC2 may be involved in myelination of oligodendrocyte [48] and glutamate synaptic transmission [49]. Prolonged, but not acute, treatment with rapamycin was reported to lead to interference with mTORC2 [50]. These data suggest that mTORC1 and mTORC2 might be differently modulated by rapamycin and Aβ.

Collectively, the present study demonstrates that REDD1 is required for Aβ-induced synaptic dysfunction and memory impairment. However, REDD1 is not involved in the process of Aβ generation and metabolism.

## 4. Materials and Methods

### 4.1. Animals

CD-1 mice weighing 25–30 g (male, 6 weeks old) were purchased from Samtako (Osan, Korea). The mice were habituated to the living environment for 1 week before each experiment. Experiments were started with 7 week old mice. Mice had freely available food and water, and were bred in a space with a 12/12 h dark/light cycle. Animals were raised according to National Institutes of Health (NIH) guidelines for the care and use of laboratory animals (NIH publications no. 8023, revised 1978), and all experiments were approved by the Institutional Animal Care and Use Committee at Dong-A University (DIACUC-approve-20-5, 20 May 2020).

### 4.2. Materials

Rabbit anti-REDD1, rabbit anti-GAPDH, rabbit antiphosphorylated extracellular signal-regulated kinase (pERK), rabbit anti-ERK, mouse anti-mTOR, and rabbit anti-Akt antibodies were purchased from Santa Cruz Biotechnology (Santa Cruz, CA, USA). Rabbit antiphosphorylated Src family kinase (pSFK), rabbit anti-Fyn, rabbit anti-pS6, and rabbit anti-S6 antibodies were purchased from Cell Signaling Technology (Beverly, MA, USA). Aβ_1–42_ was purchased from AnaSpec (San Jose, CA, USA). REDD1 shRNA (m) lentiviral particles were purchased from Santa Cruz Biotechnology (sc-45807-V, Santa Cruz, CA, USA). Anisomycin, actinomycin D, PP1, U0126, SL0101-1, and MPEP were purchased from Tocris Bioscience (Ellisville, MO, USA). 6-(1,3-Dioxo-6-(piperidin-1-yl)-1H-benzo[de]isoquinolin-2(3H)-yl)hexanoic acid (REDD1 inducer) was purchased from Sigma-Aldrich (St. Louis, MO, USA) [23]. All other materials were obtained from normal commercial sources and were of the highest grade available.

### 4.3. Aβ_1–42_ Preperation and Injection

We added 1.0% NH_4_OH directly to the Aβ_1–42_ (35–40 μL to 0.5 mg peptide or 70–80 μL to 1 mg peptide). This solution was immediately diluted with 1X phosphate-buffered saline (PBS) to a concentration of 1 mg/mL. The solution was gently vortexed and sonicated at room temperature until fully miscible. Aβ_1–42_ (10 μM) was incubated at 37 °C for 24 h to obtain various soluble oligomeric species, and 5 μL of Aβ or vehicle (PBS) was then acutely injected into the left lateral ventricle by hand under isoflurane anesthesia (induction 3% and maintenance 2%) [51]. Experiments started 7 days after the injection.

### 4.4. REDD1 shRNA Injection

REDD1 shRNA (m) lentiviral particles were bilaterally injected into the hippocampal fissure layer. Mice were placed in a stereotaxic frame (David Kopf Instruments, Tujunga, CA, USA) under isoflurane anesthesia (induction 3% and maintenance 2%). Target injection site coordinates were as follows: AP, 2.0 mm; ML, ±1.25 mm; DV, 1.75 mm [52]. Injections were performed using a 5 μL Hamilton syringe operated by a Harvard Apparatus Pump II Dual Syringe micropump. Needles were left in place for an additional 60 s to allow for the fluid to diffuse. Each side was injected individually, one immediately after the other, with 2 μL/side of REDD1 shRNA (m) lentiviral particles (1 × 10^7^ in 2 μL) at a rate of 0.2 μL/min.

### 4.5. Immunohistochemistry for REDD1

Mice were anesthetized using isoflurane (3%) at 24 h after Aβ injection, and perfused transcardially with 100 mM phosphate buffer (pH 7.4), followed by ice-cold 4% paraformaldehyde. Brains were removed and postfixed in phosphate buffer (50 mM, pH 7.4) containing 4% paraformaldehyde overnight. Brains were immersed in a solution containing 30% sucrose in 50 mM phosphate-buffered saline (PBS) and stored at 4 °C until sectioning. Frozen brains were coronally sectioned on a cryostat at 30 μm, and sections including the hippocampal area (from −1.50 mm posterior to the bregma as defined in the mouse brain atlas) were stored in a storage solution at 4 °C.

Free-floating sections (thickness, 30 μm) were incubated for 24 h in PBS (4 °C) containing rabbit anti-REDD1 (1:500 dilution), 0.3% Triton X-100, and 1.5% normal serum. Sections were incubated for 90 min with FITC-conjugated secondary antibody (1:1000 dilution). Lastly, the stained brain sections were mounted onto glass slides using Richard–Allan Scientific mounting medium (Thermo Scientific, Waltham, MA, USA). Images of histochemical samples were obtained with a Zeiss LSM 700 (Carl Zeiss AG, Oberkochen, German), and images were analyzed using ImageJ software (NIH, Bethesda, MD, USA). For the analysis of REDD1 immunoreactivity, the CA1 regions of hippocampal tissue were quantified. Quantification of REDD1 immunoactivity was performed by determining the percentage of fluorescence intensity using ImageJ software (NIH, Bethesda, MD, USA).

### 4.6. Acute-Hippocampal-Slice Preparation

Artificial cerebrospinal fluid (ACSF) was composed of NaCl (124 mM), KCl (3 mM), NaHCO_3_ (26 mM), NaH_2_PO_4_ (1.25 mM), CaCl_2_ (2 mM), MgSO_4_ (1 mM), and D-glucose (10 mM). We rapidly isolated the mouse hippocampus and submerged it in chilled ACSF. For tissue slicing, we used McIlwain tissue chopper. Hippocampal slices of 400 μm thickness were incubated in ACSF (20–25 °C, 2 h) before the experiment.

### 4.7. Western Blot

Acute hippocampal slices were used for mechanism studies. To see the effect of Aβ on REDD1 production and signaling, acute hippocampal slices were incubated with Aβ (10 μM)-containing ACSF for 4 h. For the blocking test, acute hippocampal slices were incubated with drug (inhibitors)-containing ACSF for 30 min, and then were incubated with Aβ (10 μM) + drug-containing ACSF for 4 h further. After incubation, the hippocampal slices were homogenized in ice-cold homogenize buffer (0.32 M sucrose, 1 mM EDTA, 1 mM EGTA, 1 mM PMSF, 1 mM sodium orthovanadate, one protease inhibitor cocktail tablet (Roche, Seoul, Korea) per 50 mL of buffer in 20 mM Tris-HCl buffer (pH 7.4)). Proteins from the lysates were quantified using a BCA protein assay kit. Proteins (100 μg for caspase-3 or 30 μg for others) were subjected on SDS-PAGE gels for electrophoresis and transferred to PVDF membranes at 300 mA for 2 h at 4 °C in transfer buffer (25 mM Tris-HCl (pH 7.4) containing 192 mM glycine and 20% *v*/*v* methanol). The Western blots were then incubated for 1 h with a blocking solution (2% BSA or 5% skim milk), then with primary antibodies overnight at 4 °C, washed ten times with Tween20/Tris-buffered saline (TTBS), incubated with a 1:2000 dilution of horseradish peroxidase-conjugated secondary antibodies for 2 h at room temperature, washed ten times with TTBS, and finally developed by enhanced chemiluminescence (Amersham LifeScience, Arlington Heights, IL, USA).

### 4.8. Electrophysiology

Field excitatory postsynaptic potential (fEPSP) was recorded in the CA1 area (Schaffer collateral–commissural pathway) of the acute hippocampal slices. Constant stimuli were delivered through stimulating electrode (0.033 Hz). The slope of the evoked fEPSP was averaged over consecutive recordings evoked at 30 s intervals. 30 min after the initiation of a stable baseline, high-frequency stimulation (HFS: 2 trains of 100 pulses at 100 Hz with 30 s interval) was introduced to induce long-term potentiation (LTP). LTP was quantified by comparing the mean fEPSP slope at 80 min after the TBS with the mean fEPSP slope during the baseline period. To test the effect of the REDD1 inducer or Aβ on hippocampal LTP, acute hippocampal slices were incubated with REDD1 inducer (10 μM) or Aβ (10 μM) before recording (Figure 3A,C). To test the effect of REDD1 shRNA on Aβ-induced hippocampal LTP deficits, REDD1 shRNA was injected into the hippocampi of the mice, and acute hippocampal slices were prepared from the mice 7 d after the injection. Acute hippocampal slices were incubated with Aβ (10 μM) for 4 h before recording (Figure 3D).

### 4.9. Passive-Avoidance Test

To test the effect of REDD1 shRNA on Aβ-induced memory deficit, REDD1 shRNA was injected into the hippocampi of mice, and Aβ was injected into the lateral ventricles of the mice 7 d after the shRNA injection. Passive avoidance started 7 d after the Aβ injection. The passive-avoidance box was composed of 2 rooms, namely, a dark and an illuminated room, which were separated with a guillotine door. In a training session, a mouse was located in the illuminated room, and the guillotine door opened 10 s later. When the mouse crossed the guillotine door and entered the dark room, the door closed, and 0.5 mA of electric shock was delivered through the grid floor. The next day, the mouse was relocated to the illuminated room and the guillotine door opened 10 s later. Latency time to enter the dark room was measured by 300 s. Behavioral tests and quantification were performed by investigators blind to the groups.

### 4.10. Object-Recognition Test

One day after the passive-avoidance test, the mice were habituated to the open field (25 × 25 × 25 cm) with an internal cue on one of the four walls for 10 min. Thirty minutes after habituation, the mice were re-placed in the same box with two distinct objects. The objects consisted of a glass box and a plastic cylinder. Mice were allowed to freely explore the objects for 10 min. After 2 h, mice were placed back into the same box for the test phase. The two objects were again present, but one object was now displaced to a novel one (metal ball). Mice were allowed to freely explore the environment and the objects for 5 min. Time spent exploring the displaced and nondisplaced objects was measured using video-based Ethovision XT System (Noldus, Wageningen, The Netherlands). Behavioral tests and quantification were performed by investigators blind to the groups.

### 4.11. Statistics

All statistical analyses and graphs were performed using GraphPad Prism version 5.0 (GraphPad, San Diego, CA, USA). All in vitro experiments were repeated three times. For multiple comparisons, data were analyzed by one-way analysis of variance (ANOVA) followed by Turkey’s test for significance between groups. The *t* test was only used for comparison between the two groups. Data are expressed as means ± SD with raw data. Statistical significance was set at *p* < 0.05.

## Abbreviations

AD	Alzheimer’s disease
Aβ	Amyloid β
REDD1	Regulated in development and DNA damage response 1
TSC	Tuberous sclerosis complex
ERK	Extracellular signal regulated kinase
SFK	Src family kinase
PBS	Phosphate-buffered saline
ACSF	Artificial cerebrospinal fluid
TTBS	Tween20/Tris-buffered saline
fEPSP	Field excitatory postsynaptic potential
HFS	High-frequency stimulation
LTP	Long-term potentiation
